# PROTECT: Protein circadian time prediction using unsupervised learning

**DOI:** 10.1016/j.isci.2025.113605

**Published:** 2025-09-22

**Authors:** Aram Ansary Ogholbake, Qiang Cheng

**Affiliations:** 1 Department of Internal Medicine and Department of Computer Science, University of Kentucky, Lexington, KY, USA

**Keywords:** Protein, Biocomputational method, Neural networks, Proteomics

## Abstract

Circadian rhythms regulate human physiology and their disruption is associated with diseases like Alzheimer’s disease (AD). Most proteomic datasets lack time labels and face challenges such as small samples, high dimensionality, and noise, hindering circadian analysis. We introduce PROTECT, an unsupervised deep learning method that predicts circadian sample phases without requiring time labels or known rhythmic proteins. Using greedy layer-wise pre-training and cosine-based fine-tuning, PROTECT achieves high accuracy on time-labeled datasets. Applied to unlabeled human proteomic data from postmortem brain regions and urine, PROTECT uncovers circadian disruptions in AD, identifying proteins with retained, lost, or gained rhythmicity. Proteins which retained rhythmicity show region-specific phase shifts and amplitude changes. Enrichment analysis of proteins with altered rhythmicity offers functional insights. This study systematically compares circadian patterns between AD and control subjects using proteomic data, revealing key insights into AD-related circadian dysregulation.

## Highlights

• PROTECT, an unsupervised learning model, predicts circadian phases in proteomic data
• Reveals circadian differences between control and Alzheimer’s disease subjects
• Potential drug targets and ultradian proteins found in temporal cortex brain region
• Available as an open-source code for circadian biology research

## Graphical abstract

**Figure:**
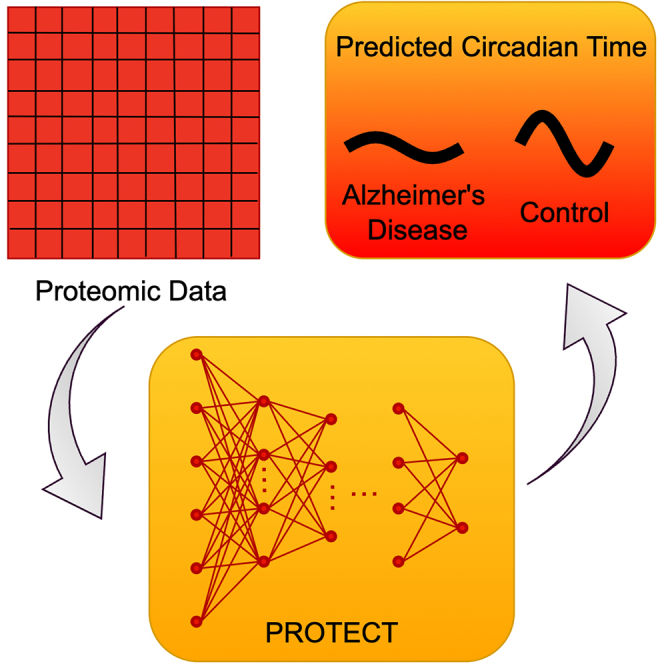

## Introduction

Circadian rhythms, driven by an internal molecular clock, regulate physiological, and behavioral processes. Disruptions to these rhythms have been associated with various pathologies, including type 2 diabetes, obesity, and neurological disorders like Alzheimer’s disease (AD).^1^^,^^2^^,^^3^^,^^4^^,^^5^^,^^6^ Hence, studying these rhythms is crucial due to their significant impact on health and disease.

In mammals, cell-autonomous circadian rhythms are driven by interconnected transcriptional-translational feedback loops. Additionally, substantial contributions to circadian processes come from posttranslational and posttranscriptional regulation.^7^^,^^8^ While the majority of research in the field of chronobiology has focused on the rhythmic expression at the mRNA level, understanding circadian rhythmicity at the protein level remains limited. This limitation is noteworthy given the acknowledged contribution of post-transcriptional mechanisms to circadian rhythms at the protein level.^9^^,^^10^

Obtaining precise sample times is currently essential for analyzing circadian rhythms in proteomic data. However, many datasets, particularly human samples, often lack labeled timestamps due to constraints imposed by health risks in collection protocols or the inability to collect precise sample times (e.g., time of death). This lack of time labels poses an urgent need for computational methods to predict the phase of each sample. Challenges arise in developing such methods due to several factors. Firstly, these datasets often contain small sample sizes with high dimensionality and significant noise. Moreover, the presence of proteins with periods shorter than 24 h, i.e., ultradian rhythms, adds complexity. These challenges cannot be effectively tackled using conventional statistical or machine learning methods. The rapid advances in deep learning techniques offer promise for overcoming these challenges and estimating the circadian phase of each sample. While efforts have been made at the mRNA level,^11^^,^^12^^,^^13^^,^^14^^,^^15^^,^^16^^,^^17^ there is a notable gap in research concerning the prediction of circadian phases in proteomic data.

As mentioned earlier, many datasets lack labeled timestamps. ZeitZeiger,^14^ TimeSignature,^12^ TimeTeller,^15^ tauFisher,^16^ and PLSR^17^ employ supervised learning approaches that require time labels and, therefore, cannot be used in practice when time information is unavailable. In contrast, we introduce an unsupervised learning approach which eliminates the need for time labeled samples to estimate circadian phases. This makes it a valuable alternative for circadian rhythm analysis, particularly in scenarios where accurate time annotations are lacking.

On the other hand, existing unsupervised or mathematical sample phase estimation (or temporal ordering) methods for gene expression data, such as CYCLOPS^11^ and CIRCUST,^18^ were specifically developed for gene expression datasets (We also tested a program of unpublished ESOCVD,^19^ which could not execute.) These methods explicitly require the use of expressions of a set of “seed rhythmic genes” to operate. The seed genes are set of known circadian rhythmic genes, including core clock genes, in animal tissues. Without these pre-selected seed rhythmic genes, these phase estimation methods may not function properly, limiting their applicability to datasets where such prior knowledge is available.

Proteomic data poses a significant challenge for applying these methods to study rhythmic proteins, as many proteins corresponding to the designated seed rhythmic genes may not be expressed in a tissue. This is because proteomic datasets typically measure the expression levels of 1,000–10,000 proteins, which is significantly fewer than the 15,000–50,000 genes typically measured in gene expression datasets. Furthermore, proteins corresponding to core clock genes are often expressed at low levels or not at all, making their measurements unavailable in proteomic datasets. Additionally, some genes known to be rhythmic at the mRNA level may not exhibit rhythmicity in their corresponding proteins, and vice versa. Previous studies have shown that only a small proportion of rhythmic proteins are rhythmic in their corresponding genes, while a large number of proteins exhibit rhythmicity even when their corresponding mRNAs do not, and vice versa.^20^

**Figure 1:**
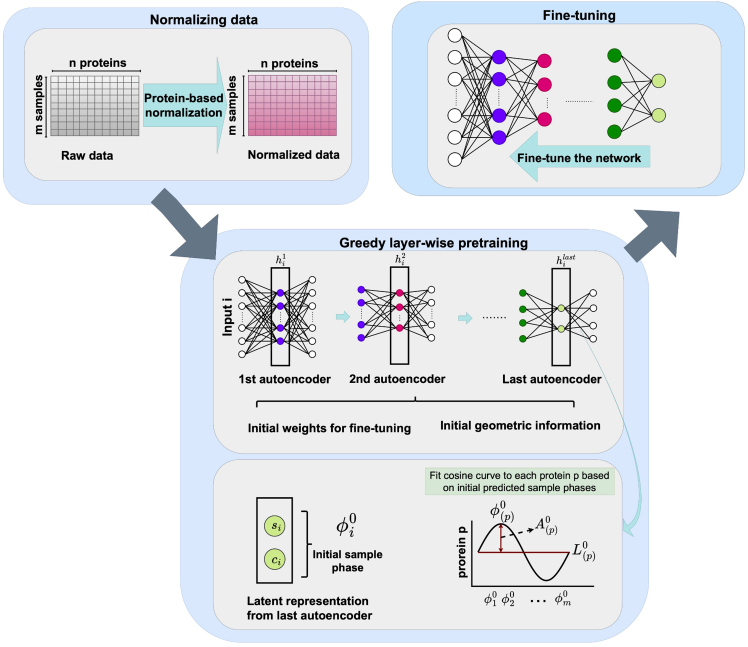
Overall diagram of the PROTECT framework for circadian phase prediction from unlabeled proteomic data The pipeline consists of three main steps: (1) Data Normalization (top-left): Raw proteomic measurements undergo protein-wise *Z* score normalization to ensure feature comparability across samples. (2) Greedy layer-wise pretraining (bottom): A deep neural network is pretrained using sequential shallow autoencoders, each learning progressively compressed representations ($h_{i}^{1},h_{i}^{2},\ldots ,h_{i}^{\text{last}}$ for sample i). The final hidden representation $h_{i}^{\text{last}}=\left(s_{i},c_{i}\right)$ is used to compute an initial phase estimate $\phi_{i}^{0}=\arctan\left(s_{i}/c_{i}\right)$, which then helps fit cosine curves to each protein p, extracting amplitude ($A_{p}^{0}$), acrophase ($\phi_{p}^{0}$), and mesor ($L_{p}^{0}$) as initialization parameters. (3) Fine-tuning (top-right): The entire network undergoes optimization with a specialized loss function that fits protein expression profiles to cosine functions, leveraging the geometric parameters to accurately predict circadian phases.

To meet the urgent need and challenges, we introduce an unsupervised deep learning approach called PROTECT (PROTEin Circadian Time prediction). PROTECT is a rhythmicity-aware model designed to predict the circadian phase of each sample in proteomic data, without requiring time labels or prior knowledge of rhythmic markers. This method can effectively handle small sample size datasets and ultradian proteins. It does not require any known seed rhythmic proteins or genes and can handle considerable noise levels present in proteomic data. PROTECT’s methodology consists of three main steps, as depicted in Figure 1. First, it normalizes the data using *Z* score normalization. Second, it pretrains the deep neural network using shallow autoencoders to compute initial circadian phase estimates and the proteins’ initial geometric information. At last, the entire network is fine-tuned by fitting proteins to cosine curves. We demonstrate the efficacy, accuracy, and robustness of our approach using mouse, Ostreococcus tauri cell, plant, fungus, and human datasets, where time labels are available. Subsequently, we investigate circadian rhythms in human datasets obtained from different brain regions and urine samples from both control and AD subjects.

Proteins, along with the metabolic pathways they modulate, often serve as the ultimate biological effectors of AD genetic.^21^ Despite the increasing body of work in recent years on discerning disparities in proteomic data between AD and control subjects,^22^^,^^23^^,^^24^^,^^25^^,^^26^ there is a lack of investigation in identifying differences between control and AD individuals based on circadian rhythms. Our developed approach can uncover AD-associated rhythmic proteins and distinguish differences in rhythmic patterns between control and AD subjects, filling this gap.

In brief, the contribution of our paper includes but is not limited to:

• We present a unique approach for accurately predicting circadian phases in un-labeled proteomic datasets, which, to our knowledge, no existing methods in the field address. It does not rely on prior information about circadian rhythmic genes or proteins, marking a significant advancement in the field of computational inference from proteomic data.
• Our method shows high prediction accuracy while effectively handling datasets with varying sample sizes, high dimensionality, and substantial noise. Additionally, it identifies ultradian rhythmic proteins, showcasing its versatility.
• Applying our method to un-labeled human proteomic datasets reveals circadian rhythmic differences between control and AD subjects across three postmortem brain regions and urine samples. This highlights the potential of our approach to uncover key insights into circadian disruptions associated with AD in proteomic datasets.

## Results

To assess the efficacy of PROTECT, we performed computational experiments on multiple public datasets. We verified the accuracy of our method using labeled proteomic datasets from human, mouse, plant, and cell models. Subsequently, we conducted experiments on unlabeled human datasets, including postmortem brains and urine. Our investigation on unlabeled human datasets focused on comparing differences between control and AD subjects. This comprehensive, multifaceted approach enabled us to evaluate PROTECT’s capabilities on different species and varying sample-size datasets.

### Hyperparameters and model details

Our model was trained using PyTorch Lightning,^27^ using a deep network with 5 hidden layers. The choice of number of layers for our network was made by a series of architectural experiments aimed at optimizing generalizability. The neuron counts of the layers ranged from $2^{\left\lfloor \log_{2}\left(f\right)\right\rfloor }$ to 2, where f is the number of features in each dataset. We tested multiple configurations and found that an architecture with 5 hidden layers comprising these neuron counts consistently yielded the best performance across a wide variety of data. This depth allowed the model to effectively capture nonlinear patterns in the proteomic features. The high dimensionality of the input, reflecting the number of proteins, required a network with sufficient depth to learn hierarchical representations. During pre-training, optimizers such as SGD, Adam, and learning rate-free methods (DAdapt-SGD and DAdapt-Adam^28^) were tested. We found that the specific optimizer did not notably impact the end results, allowing flexibility in optimizer choice for each layer. During pre-training, the first 5 AEs were trained using 7 epochs and the last AE was trained for 20 epochs. During fine-tuning, the entire network was trained for 20 epochs. To handle noise, we retrained the network for 200 epochs using the datasets with outliers removed; see Table 1 for more details of the implementation.

**Table 1:** Details for pre-training and fine-tuning stages

Training stage	Optimizer	Best learning rate	Momentum	Weight initialization
Pre-training (1st-5th AE)	Adam	Adam: 0.001	SGD: 0.85	Xavier uniform
	SGD	SGD: 0.1		
	DAdapt-SGD	DAdapt-SGD: –		
Pre-training (last AE)	DAdapt-SGD	learning rate-free	–	Xavier uniform
Fine-tuning	DAdapt-SGD	learning rate-free	–	Transferred weights

### Labeled proteomic data experiments

#### Performance evaluation

We first demonstrate the time of day prediction accuracy of PROTECT on time-labeled datasets, employing ROC curves that show the fraction of correctly predicted sample phases relative to the size of errors.^12^ Moreover, we show scatterplots of predicted sample phases with respect to the ground truths.

**Figure 2:**
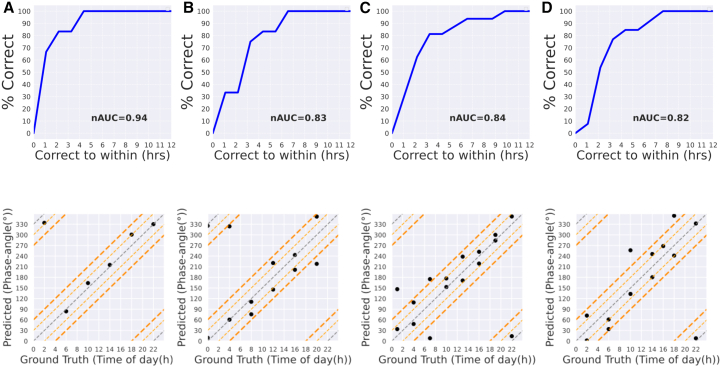
Performance and prediction accuracy of PROTECT across multiple datasets Accuracy of PROTECT on (A) Ostreococcus tauri, (B) Mouse hip articular cartilage, (C) Mouse liver, and (D) Human plasma. The top row shows ROC curves where the *y* axis shows the fraction of correctly predicted samples, and the *x* axis shows the size of errors. The bottom row shows the scatterplots of predictions vs. ground truth. Dashed orange lines indicate an error range of ±2 and ±4 h around the true time.

PROTECT shows excellent performance on various datasets, achieving a high normalized area under the curve (nAUC) of 94% on Ostreococcus tauri cell data (Figure 2A), and over 80% on mouse hip articular cartilage (Figure 2B), mouse liver^20^ (Figure 2C), and human plasma (Figure 2D) proteomic datasets. The bottom row of Figure 2 demonstrates accurate phase predictions compared to ground truth across all datasets. In particular, all samples of the Ostreococcus tauri cell data show minimal phase prediction errors, while samples in other datasets typically deviate by no more than 4 h (60° in circadian phase).

We further evaluated the performance of PROTECT on an additional mouse liver dataset,^10^ mouse brown adipose tissue (BAT), Arabidopsis thaliana plants, and Neurospora crassa fungus, with the corresponding results presented in the Supplementary Material (Figure S1). The results further demonstrate PROTECT’s ability to accurately predict sample phases.

**Figure 3:**
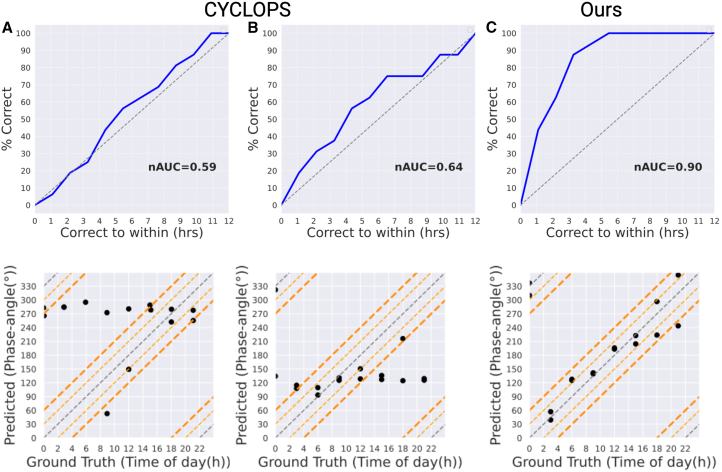
Comparison of CYCLOPS and our method on mouse liver dataset (A) CYCLOPS ROC curve and predicted sample phases vs. ground truths without using proteins corresponding to seed genes. (B) CYCLOPS ROC curve and predicted sample phases vs. ground truths after using proteins corresponding to seed genes. (C) Our ROC curve and predicted sample phases vs. ground truths. Dashed orange lines indicate an error range of ±2 and ±4 h around the true time.

We tested existing methods, originally designed for gene expression data, on the proteomic mouse liver dataset (PXD003818),^29^ which includes 16 samples and 5,301 proteins. This dataset contains the most proteins corresponding to seed rhythmic genes compared to other datasets. Figure 3A shows the ROC curve and the sample phase estimations compared to the time labels using CYCLOPS^11^ without utilizing proteins corresponding to the seed rhythmic genes. This plot shows that without using proteins corresponding to seed genes, the prediction is close to random guessing with an nAUC of 59%. Moreover, correct estimates should ideally be on the dotted diagonal gray lines (where lines in the corners account for circadian periodicity). However, most samples are predicted to have similar values. Using the available 1,076 proteins corresponding to the CYCLOPS-designated 8,504 seed rhythmic genes, the ROC curve and scatterplot in Figure 3B show that most sample phase predictions still have similar values, with the nAUC improving by only 5%, indicating CYCLOPS’s poor performance on proteomic data (More results comparing PROTECT and CYCLOPS on both proteomic and transcriptomic datasets are provided in Table S1). CIRCUST failed to execute due to the lack of core clock genes for their “synchronization procedure,” as pointed out by a CIRCUST author via GitHub discussions. In contrast, our proposed approach (PROTECT) in Figure 3C shows high accuracy, with a high nAUC of 90% on this mouse liver dataset.

We also compared PROTECT with the existing supervised methods TimeSignature and ZeitZeiger on this proteomic mouse liver dataset^29^ and a transcriptomic mouse liver dataset.^30^ For the supervised methods, we performed random train-test splits on both datasets. Since the transcriptomic dataset had a larger sample size, we applied an 80/20 split to allocate more samples for training, thereby improving generalization performance on the test set. For the smaller proteomic dataset, we used a 70/30 split to retain a sufficient number of test samples for more meaningful evaluation. To ensure fair comparison, we used the same training and test splits for both TimeSignature and ZeitZeiger. As PROTECT is unsupervised and does not require time labels for training, we applied it directly to the test samples used for evaluating the supervised methods. Each supervised method was trained three times with different random splits, and all three methods were evaluated on the corresponding test sets to account for variability. We report the average nAUC across these runs in Table S2. The results confirm that PROTECT achieves performance comparable to, and in some cases superior to, TimeSignature and ZeitZeiger.

It is also important to note that supervised methods, such as TimeSignature and ZeitZeiger are generally impractical for small-sample proteomic datasets. These methods rely on a sufficient number of time-labeled training samples to learn accurate models. However, in many proteomic studies, including those used in this work, the number of available samples is often extremely limited. For example, datasets with only 6, 12, or 13 total samples are common. In such cases, supervised methods face serious overfitting risks: with so few samples, cross-validation or train-test splits leave insufficient data for either reliable training or meaningful evaluation. In contrast, PROTECT does not require time labels and can operate directly on small unlabeled datasets, making it a more suitable and robust approach for circadian time prediction in proteomics.

Moreover, we used this mouse liver data to evaluate the robustness of PROTECT when working with fewer samples. We randomly subsampled the data by removing 3, 6, 9, and 12 samples. This process was repeated 4 times for each subsampling scenario. PROTECT was then applied to predict the sample phases, and the average results across the 4 repetitions are shown in Figure S2. While the AUC of predictions decreases slightly compared to using the full dataset, it remains consistently above 80% across all sampling levels. This robustness highlights PROTECT’s ability to perform reliably even with reduced sample sizes, underscoring its utility for sparse high-throughput proteomics data.

#### Circadian rhythmicity in known rhythmic proteins

**Figure 4:**
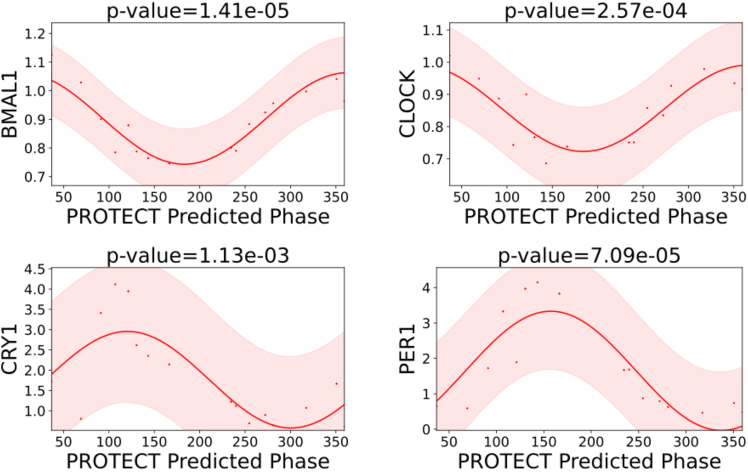
Plots of four core clock proteins in mouse liver using predicted phases by PROTECT The *y* axis represents protein expression levels, and the *x* axis represents the predicted phases (in degrees) as determined by PROTECT. Shaded areas are 95% confidence intervals. *p*-values indicate significance of the rhythmicity.

**Figure 5:**
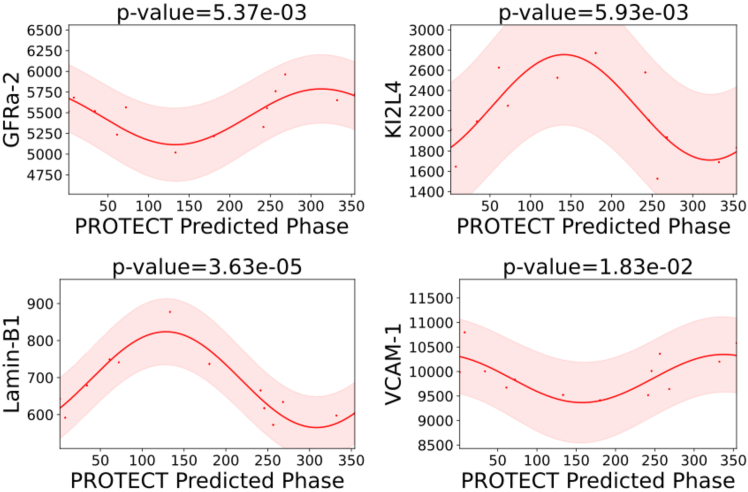
Plots of four randomly chosen proteins known to be strongly regulated by circadian cycle in human plasma using predicted phases by PROTECT The *y* axis represents protein expression levels, and the *x* axis represents the predicted phases (in degrees) as determined by PROTECT. Shaded areas are 95% confidence intervals. *p*-values indicate significance of the rhythmicity.

In Figure 4, we demonstrate the results of plotting four of the core clock proteins’ values versus the predicted sample phases using PROTECT across the mouse liver dataset^29^ where the core clock proteins are available. These results highlight the rhythmicity of these proteins using PROTECT’s predicted sample phases. Figure 5 also depicts four proteins, randomly selected from a set of proteins known to be strongly regulated by the circadian cycle in human plasma.^31^ Our predicted sample phases clearly reveal the circadian rhythmic patterns in these proteins as well. Additionally, we evaluated PROTECT’s performance on human thyroid tissue.^32^ To validate the predicted phases, we examined some known rhythmic proteins in thyroid tissue.^33^ Our results (Figure S3) confirmed the rhythmicity of these proteins, supported by significant *p* values, further demonstrating PROTECT’s reliability in detecting circadian patterns in human samples.

#### Outlier handling

Here, we show the counts of proteins and samples outliers on our time-labeled datasets. Our algorithm found no outlier samples, suggesting that all residuals for samples are similarly distributed. However, each dataset exhibited a number of outlier proteins. We excluded the outliers from each dataset and retrained the network to handle these protein outliers. We then fitted Cosinor curves against the ground truth and verified that these proteins did not exhibit significant rhythmicity at a *p* value threshold of 5e-2. We have provided a detailed summary of labeled datasets including the number of outliers removed in each dataset in Table 2.

**Table 2:** Summary of labeled datasets used to evaluate PROTECT, including sample size, time points, number of proteins used in each dataset after possible dimension reduction (proteins (before)) and after outlier removal (proteins (after)), and normalized AUC (nAUC) performance

Dataset	Species	Tissue	Samples	Time Points	Proteins (Before)	Proteins (After)	nAUC (PROTECT)
Ostreococcus tauri	Alga	Culture	6	6	855	840	0.94
Mouse cartilage	Mouse	Hip cartilage	12	6	1829	1816	0.83
Mouse liver (Mauvoisin)	Mouse	Liver	16	8	2783	2741	0.84
Mouse liver (Wang)	Mouse	Liver	16	8	2312	2312	0.90
Mouse liver (Robles)	Mouse	Liver	16	8	186	186	0.80
Mouse BAT	Mouse	Brown adipose tissue	24	12	1357	1357	0.78
Human plasma	Human	Plasma	13	7	1129	1108	0.82
Arabidopsis thaliana	Plant	Phospho-sites	6	6	4300	4287	0.88
Neurospora crassa	Fungus	Whole cell	24	12	516	516	0.87

### Un-labeled proteomic data experiments

#### Data preparation for control and AD subjects comparison

In preparing data for comparing control and AD subjects in unlabeled brain datasets, we took several steps. Initially, we removed proteins with missing values from both control and AD datasets. To ensure a fair comparison, we then used the common set of proteins in both groups. Moreover, to handle different sample sizes in control and AD groups, particularly in TC and DLPFC datasets, we made them equal by matching age and sex characteristics. In the urine dataset with numerous missing values, we removed proteins with missing values in over 50% of samples, following a similar approach as in the original paper.^34^ Then, we found the intersection of all proteins in control, MCI, and AD subjects. On the unlabeled dataset, similar to the time-labeled datasets, we removed the outlier proteins. See Table 3 for the detailed summary of unlabeled dataset.

**Table 3:** Summary of unlabeled human datasets, including sample sizes and the number of proteins before and after outlier removal for each group

Dataset	Tissue	Samples (Control/AD/MCI)	Proteins (Before)	Control (After)	AD (After)	MCI (After)
Temporal Cortex	Brain	29/29/–	2425	2425	2424	–
Parietal Cortex	Brain	25/25/–	3389	3389	3389	–
DLPFC	Brain	26/26/–	2483	2470	2475	–
Urine	Urine	43/43/43	555	555	555	552

#### Disparities between AD and control subjects

To assess disparities in circadian rhythm between AD and control groups, we applied the compareRhythms model^35^ to categorize proteins based on their rhythmicity status. This analysis identified proteins that either lost or gained rhythmicity in AD compared to controls, as well as proteins that remained rhythmic in both conditions. For proteins rhythmic in both conditions, compareRhythms further classified them based on whether they maintained similar phase and amplitude characteristics or exhibited differences between groups.

For proteins that maintained rhythmicity in both conditions, we used circaCompare^36^ to quantify specific differences in acrophase and amplitude between AD and control groups. This provided precise measurements of phase shifts and amplitude changes in rhythmic proteins affected by AD pathology.

**Figure 6:**
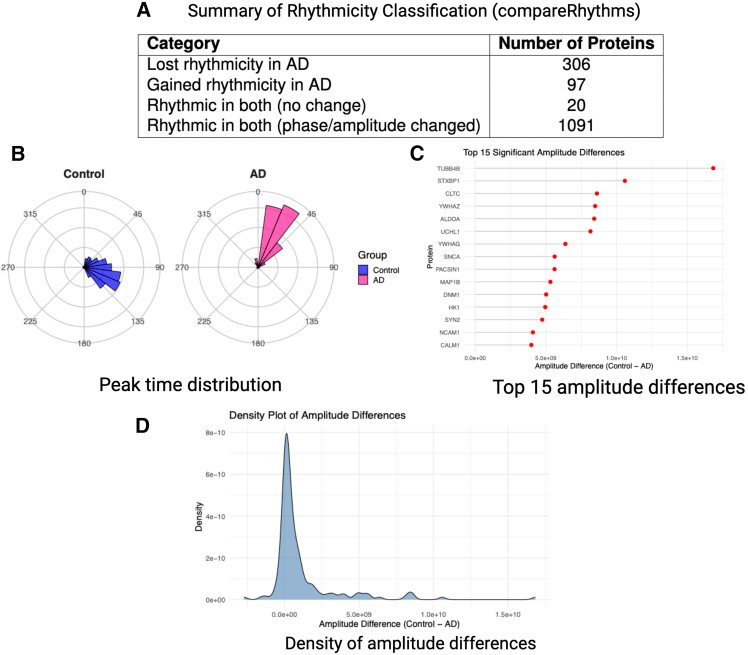
Disparities between control and AD subjects in the temporal cortex (A) Summary of rhythmicity classification from compareRhythms. (B–D) Visualization of peak time distribution, top proteins with high and significant amplitude changes, and overall amplitude difference density using PROTECT predicted phases.

Figures 6, S4, and S5 illustrate the disparities between control and AD subjects in the TC, parietal association cortex, and DLPFC brain regions. First, summary tables derived from compareRhythms are provided to demonstrate different categories of rhythmic proteins in control, AD, or both (Figures 6A, S4A, and S5A). The results indicate a higher count of proteins that lose rhythmicity in AD subjects compared to control in all brain regions. In the TC region, 306 proteins lose rhythmicity in AD and 97 gain rhythmicity in AD. In the parietal association cortex 316 proteins lose rhythmicity and 272 gain rhythmicity in AD. In the DLPFC region, fewer proteins show altered rhythmicity, with comparable numbers losing (81) and gaining (80) rhythmicity in AD. In the TC and DLPFC regions, large proportions of proteins exhibit rhythmicity. In the TC region, out of 2425 proteins, 1111 are rhythmic in both control and AD subjects. In the DLPFC region, out of 2483 proteins, 1409 are rhythmic in both control and AD subjects. However, in the parietal association cortex, lower proportions of proteins show rhythmicity. Specifically, out of 3389 proteins, 450 are rhythmic in both conditions.

Figures 6B, S4B, and S5B illustrate the distribution of peak times within a 24-h (360°) circadian cycle for rhythmic proteins whose amplitude and phase differ between control and AD subjects. To ensure consistency across datasets, we used EHD1—a protein that exhibited rhythmicity in all three brain regions and urine samples on different groups as a reference point. We set the phase of EHD1 to zero and shifted the acrophases of other proteins relative to this one, thereby providing a common baseline. Dispersed protein peak distributions arise in the parietal association cortex unlike the TC and DLPFC. In the latter two regions, the peak times of rhythmic proteins are more concentrated in smaller areas and many proteins peak around same times. Additionally, we used circaCompare to calculate the average acrophase difference between control and AD subjects on statistically significant phase differences. These phase shifts varied across brain regions. In the parietal association cortex, the mean phase difference was −4.23 h, indicating that rhythmic proteins in AD subjects peak later than in controls. In contrast, the TC and DLPFC exhibited similar shifts, with mean differences of +2.13 h and +2.51 h, respectively, suggesting that rhythmic proteins in AD tend to peak earlier compared to controls in these regions.

We then illustrate top fifteen proteins with statistically significant and highest amplitude differences between control and AD subjects for each brain region using the circaCompare program. This is depicted in Figures 6C, S4C, and S5C on TC, parietal association cortex, and DLPFC regions, respectively. As shown, in TC and DLPFC, the control condition has higher amplitude than AD in top significant proteins. However, in parietal association cortex, most of the significant proteins have higher amplitude in AD than in control which shows that these differences are brain region specific.

To explore overall amplitude alterations, we plotted density distributions of amplitude differences (calculated as Control amplitude minus AD amplitude) for proteins with statistically significant amplitude difference in each brain region (Figures 6D, S4D, and S5D). These plots revealed consistent trends with subtle regional distinctions. In the TC and DLPFC, the distributions were right-skewed, with a visibly larger density on the positive side, indicating that a substantial portion of proteins had higher amplitudes in control subjects compared to AD. In the parietal association cortex, although the density plot also shows a heavier right tail, the mean amplitude difference was negative, suggesting that the majority of proteins had slightly higher amplitudes in AD overall.

These findings highlight the differences between control and AD subjects across distinct brain regions. Identifying the underlying mechanisms driving these cortical differences in rhythmicity may open new therapeutic avenues for future exploration (to see results on urine dataset please refer to Table S3 and Figure S6).

#### Enrichment analysis

**Figure 7:**
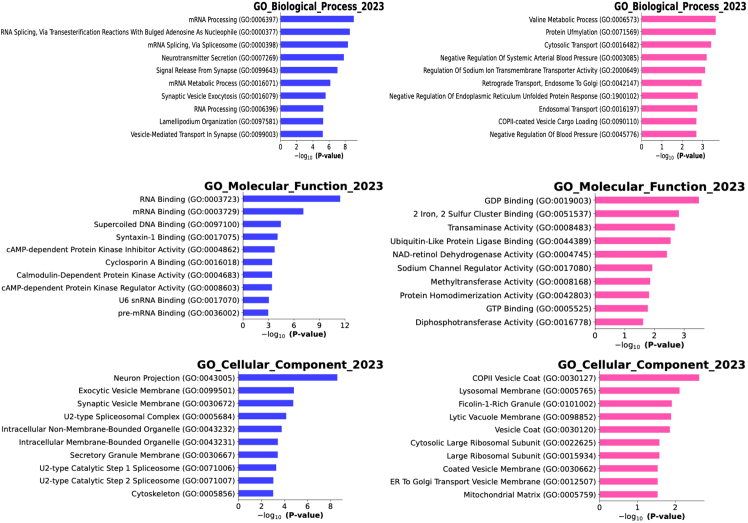
Gene ontology enrichment analysis of proteins that lose rhythmicity in AD (blue), and in proteins that gain rhythmicity in AD subjects (pink) on temporal cortex

We conducted gene ontology (GO) enrichment analysis using GSEApy,^37^ which serves as an interface between Python and Enrichr web services.^38^^,^^39^^,^^40^ The analysis was conducted on proteins that lost rhythmicity in AD (control-specific rhythmic proteins) and those that gained rhythmicity in AD (AD-specific rhythmic proteins), for each brain region (Figures 7, S7, and S8).

Proteins that lost rhythmicity in AD were significantly enriched in synaptic signaling and RNA processing pathways, including mRNA splicing via the spliceosome, neurotransmitter secretion, and vesicle-mediated transport in synapse. These processes are integral to neuronal communication and cognitive functions. Disruption in mRNA splicing has been implicated in AD, where spliceosomal proteins aberrantly aggregate and mislocalize to Tau neurofibrillary tangles, leading to splicing defects.^41^ Additionally, impairments in neurotransmitter secretion, particularly glutamate, have been associated with synaptic dysfunction in AD.^42^

In contrast, proteins that gained rhythmicity in AD showed enrichment in processes including valine metabolic process and response to endoplasmic reticulum (ER) stress. Valine metabolism has been implicated in AD pathology, with reduced serum valine associated with disease progression and tau-related neurodegeneration such as AD.^43^ In particular, valine metabolism is important for brain function as it contributes to glutamate synthesis, a key neurotransmitter implicated in cognitive processes and synaptic function. Similarly noteworthy, the ER stress response involves signaling pathways that work to restore proper protein folding in the ER, manage the protein load, and degrade misfolded proteins. The unfolded protein response is one of the main pathways managing the cellular response to ER stress. It acts to restore ER homeostasis. If ER stress is prolonged, apoptotic cell death pathways may be activated. This enrichment aligns with literature on misfolded proteins in AD.^44^

It is important to note that there is often an inherent bias in functional enrichment analysis. Our primary objective in using GSEA is not to establish definitive biological proof but rather to explore potential functional relevance of the rhythmic proteins. In this regard, the pathway analysis results should be viewed as a complementary and exploratory perspective to our modeling process, offering additional insights into the biological context of rhythmic proteins.

#### Hub proteins and related drugs

**Figure 8:**
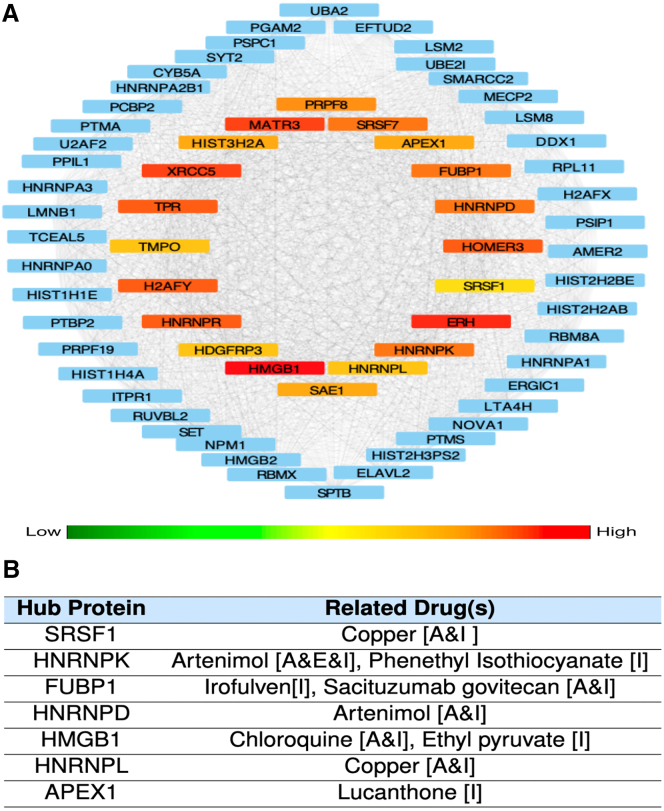
Hub proteins in temporal cortex (A) Network depiction of the first twenty hub proteins based on degrees and proteins directly interacting with these hub proteins. The colorbar indicates the connection degrees of the hub proteins. (B) Hub proteins and related drugs. The status of each drug is shown in the brackets next to it: A, I, and E indicate that the drug is approved, investigational, and experimental, respectively.

In addition, we present the top 20 hub proteins along with their directly connected first-stage nodes in the proteomic data of control-specific rhythmic proteins within the TC (Figure 8A). Similar analyses for the parietal association cortex and the DLPFC datasets are provided in Table S4. To achieve this, we initially constructed a protein coexpression network using the WGCNA R package,^45^ focusing on proteins that display rhythmicity in the control group but lose rhythmicity in AD. We applied the cytoHubba Cytoscape plugin^46^^,^^47^ and utilized the degree algorithm to identify and highlight the top-ranked proteins based on their connectivity with other proteins. Recent studies have linked some of these proteins to be therapeutic targets for AD. For instance, SRSF1 has been implicated in suppressing the formation of a CD33 splicing isoform associated with AD.^48^ Moreover, HMGB1 has been proposed as a clinical biomarker and validated as a non-invasive indicator of blood-brain barrier (BBB) dysfunction and neuroinflammation, enabling the assessment of neurodegeneration progression in both AD and MCI patients.^49^ Through the use of DrugBank,^50^ we discovered that seven of these hub proteins represent potential drug targets. The corresponding drugs for each target are shown in Figure 8B, categorized based on their development status as approved, investigational, or experimental.

### Rhythmic proteins found in each dataset

We identified rhythmic proteins through cosine curve fitting based on the predicted phases. A protein was considered rhythmic if it satisfied three criteria: (1) a Benjamini-Hochberg adjusted *p* value (FDR) less than 0.05, (2) a relative amplitude (rAMP, defined as the ratio of amplitude to mesor) of at least 0.1, and (3) a coefficient of determination ($R^{2}$) of at least 0.1. This enabled robust detection of rhythmic patterns of proteins. The full list of rhythmic proteins for each dataset is provided in Data S1, File Sheet 1.

Our analysis revealed both shared and tissue-specific rhythmic proteins across datasets. In total, 368 proteins were found to be rhythmic in three or more datasets (Data S1, File Sheet 2), suggesting a set of broadly conserved rhythmic proteins. Among the three brain control regions, over 270 proteins were consistently rhythmic across all three regions in control subjects, indicating the presence of a robust core circadian proteome in the human brain (Data S1, File Sheet 3). Additionally, we identified 206 proteins that exhibited rhythmicity in both brain regions and urine samples (Data S1, File Sheet 4), suggesting systemic circadian regulation that extends beyond the central nervous system.

To provide comparative context, we also analyzed mouse tissue datasets, which revealed more tissue-specific rhythmicity patterns than observed in human samples. Each mouse tissue had its own unique set of rhythmic proteins, with relatively limited overlap between them (Data S1, File Sheet 5).

### Ultradian proteins

In the TC dataset, we utilized predicted sample times to identify potential ultradian rhythmic proteins. Our selection criteria included FDR <5e−4, rAMP ≥0.2, and $R^{2}\geq 0.6$ to select the proteins with a period of 12 h. Through this analysis, we identified six proteins (ACOT7, CLTA, ELMO1, MDP1, NIT1, and SH3GL2) exhibiting these characteristics, as illustrated in Figure S9.

## Discussion

To study circadian rhythms at the protein level, sample times are needed. However, proteomic datasets, especially in humans, often lack explicit time labels. Other challenges include: the sample sizes are typically very small; ultradian proteins typically exist; and knowledge of rhythmic proteins are limited. To address these issues, PROTECT has been designed to predict sample phases in proteomic data in an unsupervised manner. This method is appropriate for different sample sizes and does not require *a priori* information. The effectiveness of PROTECT was validated through testing on time-labeled datasets, achieving remarkable results with over 80% nAUC on all instances. Our exploration of un-labeled human datasets using PROTECT’s time predictions revealed differences between control and AD subjects in three brain regions and urine samples. Additionally, the study provides potential drug targets and identifies some ultradian rhythmic proteins in TC.

PROTECT is generalizable to other circadian omics data types, such as transcriptomic and metabolomic datasets,^20^^,^^30^^,^^51^^,^^52^^,^^53^^,^^54^^,^^55^^,^^56^^,^^57^ as these data types share similar periodic structures. Unlike existing methods tailored for transcriptomics, PROTECT does not rely on time information or predefined rhythmic features, such as known rhythmic genes or metabolites, making it broadly applicable to these types of datasets.

To validate this, we applied PROTECT to some publicly available circadian transcriptomic and metabolomic datasets. Specifically, we analyzed transcriptomic data from a mouse liver study,^20^ which includes both proteomic and transcriptomic measurements, as well as a baboon amygdala dataset^51^ and a mouse kidney dataset.^52^ Additionally, we evaluated metabolomic data from a mouse liver study.^53^ PROTECT successfully predicted sample phases across these datasets, with detailed results provided in the Supplementary Material (Figure S10). These findings further support the robustness of our method and its potential for broader applications in circadian and diurnal research.

Our downstream analyses using compareRhythms and circaCompare allowed us to categorize proteins not only by gain or loss of rhythmicity but also by differences in phase or amplitude. These analyses revealed systematic shifts in rhythmic timing in AD—delays in some brain regions and advances in others—suggesting complex, region-specific alterations in circadian timing networks. In the TC and DLPFC, proteins in AD tended to peak earlier than in controls, whereas the parietal cortex showed later peak times in AD. Moreover, amplitude changes were more pronounced in control samples in some regions, but the opposite trend was seen in others, indicating that circadian disruption in AD is not uniform in all regions.

Functional enrichment analysis provided biological context for these findings. Proteins losing rhythmicity in AD were enriched in synaptic signaling and RNA processing pathways, both of which are intimately linked to neuronal function and cognitive performance. Conversely, proteins gaining rhythmicity in AD were enriched in metabolic processes and stress response pathways, particularly those associated with the ER. These results are consistent with prior studies linking ER stress and aberrant protein folding to neurodegenerative pathology in AD. Importantly, these enrichments were not used to establish definitive conclusions but rather to provide exploratory insight into the functional implications of the circadian alterations uncovered by PROTECT.

Further, we used network analysis to identify hub proteins among control-specific rhythmic proteins. Several of these hubs—such as SRSF1, HNRNPK, and HMGB1—have previously been implicated in neurodegenerative processes, and some have known drug interactions. This suggests that the rhythmic structure of the control-specific proteins that lose rhythmicity in AD, may intersect with actionable therapeutic targets.

In addition to circadian patterns, PROTECT successfully identified ultradian rhythmic proteins (periods shorter than 24 h), which are often overlooked in transcriptomic-focused studies. These proteins may open up new directions for studying non-circadian rhythmicity in proteomics.

### Limitations of the study

Despite its excellent performance, PROTECT requires further exploration. In our study, we did not address the impact of the postmortem interval (PMI) on postmortem human brain datasets. PMI refers to the duration between the time of death and sample collection, which can influence proteomic values. While most samples used in this paper have a PMI of less than 10 h, thus minimally impacting the study, future work should investigate the effect of PMI on predicted times.

One limitation of PROTECT lies in its strategy for handling missing values within the proteomic dataset. The current practice of excluding proteins with missing values may lead to the exclusion of valuable data that could contribute to a more comprehensive understanding of our investigation. As advances in data collection lead to fewer missing values, we expect that more proteins can be incorporated into rhythmicity analysis.

Also, to determine which protein should exhibit its peak at time 0 and subsequently arrange the remaining proteins in relation to this target protein, we employed EHD1. This protein showed rhythmicity in all datasets for both control and AD subjects. We established the peak value of EHD1 at time 0 and organized the other proteins relative to EHD1. In the future, with more studies on proteins’ peak times, we can use some known proteins as the target protein.

Besides potential future enhancements to PROTECT itself, systematically investigating these limitations provides broader understanding of the data dependencies and robustness in circadian proteomic modeling. In turn, with further investigation into these practical data constraints alongside algorithmic advances, PROTECT may significantly influence standardized protocols for circadian omics analysis, thus enabling more consistent and accurate biological findings.

## Resource availability

### Lead contact

Further information and requests for resources should be sent directly to the lead contact, Qiang Cheng (qiang.cheng@uky.edu).

### Materials availability

This study did not generate new unique reagents.

### Data and code availability

• Data: All data used in this paper were obtained from existing studies and their references are listed in the key resources table.
• Code: The code is available at: https://github.com/aramansary/PROTECT/tree/main.

## Acknowledgments

This research was supported by the National Artificial Intelligence Research Resource (NAIRR) Pilot NSF OAC 240219, and Jetstream2, Bridges2, and Neocortex Re- sources. Additional support was provided by the 10.13039/100000001NSF (IIS 2327113, ITE 2433190) and the 10.13039/100000002NIH (R21AG070909, P30AG072946, and R01HD101508-01).

## Author contributions

Conceptualization, formal analysis, investigation, methodology, software, visualization, writing – original draft: A.A.O.; conceptualization, funding acquisition, investigation, methodology, supervision, writing – review and editing: Q.C.

## Declaration of interests

The authors declare no competing interests.

## STAR★Methods

### Key resources table

**Table:** 

REAGENT or RESOURCE	SOURCE	IDENTIFIER
**Deposited data**		

Mouse hip articular cartilage	Dudek et al.^58^	https://doi.org/10.1016/j.joca.2021.02.008
Mouse liver	Mauvoisin et al.^20^; Wang et al.^29^; Robles et al.^10^	https://doi.org/10.1073/pnas.1314066111; https://doi.org/10.1016/j.cmet.2016.10.003; https://doi.org/10.1371/journal.pgen.1004047
Ostreococcus tauri cells	Noordally et al.^59^	https://doi.org/10.1093/jxb/erad290
Human plasma	Depner et al.^31^	https://doi.org/10.1073/pnas.1714813115
Mouse brown adipose tissue (BAT)	Qian et al.^60^	https://doi.org/10.1016/j.mcpro.2023.100675
Arabidopsis thaliana plants	Krahmer et al.^61^	https://doi.org/10.1016/j.mcpro.2021.100172
Neurospora crassa fungus	Hurley et al.^62^	https://doi.org/10.1016/j.cels.2018.10.014
Temporal Cortex (TC)	Johnson et al.^63^	https://doi.org/10.1038/s41591-020-0815-6
Parietal Association Cortex	Carlyle et al.^64^	https://doi.org/10.1016/j.neurobiolaging.2021.04.012
Dorsolateral Pre-frontal Cortex (DLPFC)	Beach et al.^65^; Johnson et al.^63^	https://doi.org/10.1111/neup.12189; https://doi.org/10.1038/s41591-020-0815-6

**Software and algorithms**		

Code	This paper	https://github.com/aramansary/PROTECT/tree/main
PyTorch Lightning	Falcon et al.^27^	https://github.com/Lightning-AI/pytorch-lightning
CosinorPy	Moškon^66^	https://github.com/mmoskon/CosinorPy
Python	Python	https://www.python.org/
compareRhythms	Pelikan et al.^35^	https://github.com/bharathananth/compareRhythms
circaCompare	Parsons et al.^36^	https://github.com/RWParsons/circacompare
WGCNA	Langfelder et al.^45^	https://fuzzyatelin.github.io/bioanth-stats/module-F21-Group1/module-F21-Group1.html#Weighted_Gene_Correlation_Network_Analysis
Cytoscape	Shannon etl al.^46^; Chin et al.^47^	https://cytoscape.org/
GSEApy	Fang et al.^37^	https://github.com/zqfang/GSEApy

### Method details

#### Labeled proteomic data

Several time-labeled proteomic datasets were utilized, sourced from mouse hip articular cartilage (PXD019431),^58^ three different mouse liver datasets,^10^^,^^20^^,^^29^ Ostreococcus tauri cells,^59^ human plasma,^31^ mouse brown adipose tissue (BAT),^60^ Arabidopsis thaliana plants,^61^ and Neurospora crassa fungus.^62^ Mouse hip articular cartilage was collected every four hours over two days, sampling 6 animals at each time point. We calculated the average of label-free quantification (LFQ) intensity across all six animals for each time point, resulting in a set of 12 samples. All three mouse liver datasets consist of 16 samples obtained from mice at 3-hour intervals over a 2-day span. The mouse liver data from Wang et al.^29^ and Mauvoisin et al.^20^ quantify the relative protein abundance in each of the 16 samples against a common reference sample, which was labeled using the SILAC method. The dataset mentioned in Wang et al.^29^ contains about 1000 more proteins than the one referenced in Mauvoisin et al.^20^ In the dataset from Robles et al.,^10^ the values are the median of the triplicates z-scored normalized log2 ratios. The Ostreococcus tauri cell dataset provides the mean of normalized abundance per time point, encompassing 6 samples. The human plasma data consists of samples from 6 healthy young males at different time points over two days. We utilized the time points with more than one subject and averaged them. This resulted in time points of 1, 5, 9, 13, 15, 17, and 21 for one day, and 1, 5, 9, 13, 17, and 21 for the second day. The mouse brown adipose tissue dataset includes samples collected over two days at 2-hour intervals. For the Arabidopsis thaliana dataset, proteomic measurements were taken at six time points, each spaced 4 hours apart, with five replicates per time point. We used the averaged values across replicates at each time point for analysis. The Neurospora crassa dataset contains protein abundances measured every 2 hours over a two day period. We averaged the replicates at each time point to generate a single representative time course.

#### Un-labeled proteomic data

In our exploration of circadian rhythms in unlabeld human proteomic data, we examined three brain regions: the Temporal Cortex (TC),^63^ the Parietal Association Cortex,^64^ and the Dorsolateral Prefrontal Cortex (DLPFC).^63^^,^^65^ Additionally, we incorporated a dataset derived from urine samples.^34^ In the TC and DLPFC datasets, the LFQ intensity is used for protein quantitation. In the parietal association cortex dataset, the calculation of protein intensities involves summing the TMT reporter ions corresponding to all peptides assigned to each protein. In the urine dataset, protein quantification is conducted utilizing the intensity-based absolute quantification (iBAQ) algorithm.

#### PROTECT

We developed PROTECT, an unsupervised learning method, to predict the time of high-dimensional proteomic samples based on the data itself, without relying on any *a priori* information or time labels. Proteomic data includes both rhythmic and non-rhythmic proteins. Rhythmic proteins typically exhibit periodic patterns, with values peaking at certain times of the day and dipping at others, while non-rhythmic proteins have no periodic patterns. Moreover, the peak times among rhythmic proteins are different. Without known sample times, rhythmic patterns are obscured, and PROTECT aims to recover them. PROTECT addresses key challenges in studying circadian rhythms using proteomic data, including small sample sizes, the presence of ultradian proteins, and limited knowledge of rhythmic proteins, especially in human datasets.

PROTECT utilizes a deep neural network (DNN) to predict the phase of each sample in proteomic data using a greedy layer-wise technique, inspired by Hinton et al.’s work.^67^ The DNN architecture in PROTECT is well-suited for high-dimensional data. It consists of an input layer, multiple hidden layers, and an output layer with two neurons representing a single angular phase. Training the network involves pre-training the DNN and obtaining the weights of the corresponding DNN neurons, followed by fine-tuning the weights to predict sample phases by regressing with proteins’ cosine models.

Greedy layer-wise reconstruction has advantages shown in,^67^ which we adapt for pre-training our DNN architecture. The optimized weights obtained in the pre-training stage provide an effective initialization, facilitating effective optimization for learning the sample phases and fitting downstream cosine models in the fine-tuning stage. This approach is particularly effective for small datasets because of its robust initialization of the network’s weights, which can be crucial when data is limited. Additionally, by training each layer independently, the network captures features at different levels of abstraction, reducing the risk of overfitting that often accompanies small sample sizes. The hierarchical representation further augments the network’s capability to capture intricate patterns within the high-dimensional proteomic data.

PROTECT’s methodology is structured into three main steps: Normalizing data, Greedy layer-wise pre-training, and Fine-tuning.

##### Normalizing data

In the first step, the proteomic data, which includes m samples with n protein measurements per sample, undergoes z-score normalization. This involves subtracting the mean and dividing by the standard deviation for each protein across all samples. This standardization ensures that all proteins are on a comparable scale, improving the stability and performance during the training process. The resulting normalized data is then utilized in the subsequent step: Greedy layer-wise pre-training.

For datasets with a very large number of features (e.g., n≥5000), we recommend applying a feature selection or dimensionality reduction technique after normalizing the data to enhance computational efficiency and model performance. One effective approach is to use k-means clustering, where the features are grouped into clusters, and only the clusters with the highest variance are kept. Alternatively, high-variance features can be directly selected, where only the features with the highest variance across samples are chosen.

##### Greedy layer-wise pre-training

This step involves greedily training each layer of the DNN using a separate shallow auto-encoder (AE) network suitable for datasets with small sample sizes. The primary objective is to encode the input into lower dimensions and capture intricate patterns in the proteomic data using each AE, ultimately reaching the output layer where the features represent the encoded data in two dimensions (see an example of encoded data in Figure S11).

Each shallow auto-encoder consists of an input layer, a hidden layer, and an output layer. The hidden layer’s output from the AE at layer l ($h_{i}^{l}$) of the DNN becomes the input for the AE at layer l+1 ($h_{i}^{l+1}$) in the DNN. The objective function for each AE is the mean squared error (MSE) between the input and output, providing a measure of how well it reconstructs the input.

After training the last AE, the values of the two nodes in its hidden layer ($h_{i}^{last}$), denoted as $s_{i}$ and $c_{i}$ for sample i, are extracted. These feature values compute the initial phase, $\phi_{i}^{0}$, for each sample i, as expressed by the equation:

(Equation 1) $$\phi_{i}^{0}=\arctan\left(\frac{s_{i}}{c_{i}}\right)\text{.}$$

Subsequently, CosinorPy^66^ is employed to extract geometric information for each protein using the initial predicted phases ($\phi_{i}^{0}$). This includes parameters such as amplitude ($A_{p}^{0}$), mesor ($L_{p}^{0}$), and acrophase ($\phi_{p}^{0}$), achieved by fitting each protein p to a cosine curve using the initial predicted sample phases. This geometric information is then incorporated into the objective function of the DNN in the next step, which is fine-tuning.

In summary, the pre-training process begins with training the first AE, where normalized proteomic data serves as the input. From this, we extract the hidden layer values $h_{i}^{1}$. Then, we proceed to train the second AE, utilizing the hidden layer output $h_{i}^{1}$ of the first AE as its input, and extract the hidden values $h_{i}^{2}$. This iterative process continues until we extract the features of the last AE, which yields the initial angular phase. The initial phase is used to calculate the initial geometrical information of each protein which will be used in fine-tuning. Moreover, the extracted features from each AE are then utilized to initialize the weights of the DNN in fine-tuning.

##### Fine-tuning

After pre-training the DNN using sequence of shallow AEs and obtaining the initial weights and geometrical information, we fine-tune the network to predict sample phases. In this stage, the network learns to align protein expressions with rhythmicity by fitting each protein to a cosine function. We organize the data into a table where each row corresponds to a sample, and each column represents a proteins’s expression; that is, $x_{ip}$ represents the original observation for sample i and protein p. We use a parameterized cosine function to fit the observations, given by:

(Equation 2) $${\hat{x}}_{ip}=L_{p}+A_{p}\;\cos\left(\omega_{p}{\hat{\phi }}_{i}+\phi_{p}\right)\text{,}$$

where ${\hat{\phi }}_{i}$ represents the sample phase, and $L_{p}$, $A_{p}$, and $\phi_{p}$ are the mesor, amplitude, and the acrophase of protein p, respectively. $2\pi /\omega_{p}$ represents the period of protein p. The objective function is defined as:

(Equation 3) $$L=\frac{1}{m}\sum_{i=1}^{m}\frac{1}{n}\sum_{p=1}^{n}\|x_{ip}-{\hat{x}}_{ip}{\|}_{q}^{q}+\lambda R\left(\Theta \right)\text{.}$$

Here, ${\left\|\cdot \right\|}_{q}$ is an $l_{q}$ norm with respect to both i and p, with q as a positive value. Θ represents the set of all relevant parameters (including $L_{p}$, $A_{p}$, ${\hat{\phi }}_{i}$ and $\phi_{p}$), and R(Θ) represents a regularization function on the parameters. λ is a non-negative hyperparameter balancing the fitting error and regularization.

The learnable parameters $L_{p}$, $A_{p}$, and $\phi_{p}$ are initialized with the values calculated during the pre-training step. $\omega_{p}$ allows the model to fit proteins with different rhythmicity such as circadian rhythms and ultradian rhythms. This flexibility ensures that the model accurately captures various rhythmic patterns in protein expression.

This objective function aims to find the optimal phase for each sample and determines the best geometrical parameters, ensuring an accurate and effective fitting of the protein data to the cosine curve. Moreover, by incorporating the unique geometric information of each protein in each sample, this objective function helps with handling small sample sizes (to see an example of a random protein recovering its rhythmicity during this stage, see Figure S12).

We have explored the use of multiple regularization functions including $l_{1}$ norm, $l_{2}$ norm and total variation (TV) regularization. The total variation term aims to reduce the abrupt shifts between consecutive sample phases and is defined as:

(Equation 4) $$\sum_{k=2}^{m}\left|{\hat{\phi }}_{i_{k}}-{\hat{\phi }}_{i_{\left(k-1\right)}}\right|\text{,}$$

where sample phases are sorted in ascending order, and $i_{k}$ represents the k-th sorted sample for k=1,…,m.

Our experiments with various λ values for different regularization functions on labeled datasets showed no significant improvement in phase prediction accuracy when λ>0. Therefore, we set the λ=0 in our final model. Moreover, we employed q=1 for the fitting error term.

This parametric objective function enables the predicted phases to take into account the protein geometrical information, such as amplitudes, acrophases, and periods. Moreover, it is applicable when the noise in the data is non-Gaussian.^66^^,^^68^ In addition, cosine curve fitting has been shown to be effective on data without replicates, containing outliers, irregularly spaced time intervals, and unbalanced data distributions where more samples are collected at certain times of the day.^33^^,^^66^^,^^69^ To see the performance of the objective function, refer to the convergence results provided in Figures S13–S15.

#### Screening for potential outliers

In the fine-tuning phase, we design our model to handle inherent noise and potential outliers in proteomic data. The process of detecting outliers is as follows:

• Sample-level outlier detection: For each sample, we calculate the averaged fitting error using:

(Equation 5) $$E_{i}=\frac{1}{n}\sum_{p=1}^{n}\left(x_{ip}-{\hat{x}}_{ip}\right),i=1,\ldots ,m\text{.}$$

Thus, $E_{i}$ represents the fitting residues averaged for all proteins p=1,…,n. We then compute the mean and standard deviation of these $E_{i}$ values across all samples, respectively denoted by $m_{s}$ and $\sigma_{s}$. If a sample’s deviation exceeds two standard deviations from the mean $m_{s}$, i.e., if $\left|E_{i}-m_{s}\right|>2\sigma_{s}$, then sample i is considered an outlier.

• Protein-level outlier detection: For each protein, we obtain its average fitting residual over all samples:

(Equation 6) $$D_{p}=\frac{1}{m}\sum_{i=1}^{m}\left(x_{ip}-{\hat{x}}_{ip}\right),p=1,\ldots ,n\text{.}$$

Thus, $D_{p}$ represents the fitting quality averaged for all samples i=1,…,m. We compute the mean $m_{p}$ and standard deviation $\sigma_{p}$ of $D_{p}$ across all proteins. If a protein’s deviation exceeds two standard deviations from the mean $m_{p}$, i.e., if $\left|D_{p}-m_{p}\right|>2\sigma_{p}$, then protein p is considered an outlier candidate. To ensure no significant cyclic proteins are removed, the outlier candidates undergo a second screening process. In this screening, if the predicted amplitude of a candidate falls below the 75th percentile of all predicted amplitudes, it is classified as an outlier protein.

Through this approach, we identify outliers (resp. outlier proteins) that significantly deviate from the majority of samples (resp. proteins). Subsequently, we remove the outlier samples and proteins to retrain the model. This process minimizes the influence of outlier or corrupted proteins and samples that have unusually large residuals.

### Quantification and statistical analysis

In this study, we used PyTorch Lightning to construct our unsupervised deep learning model, PROTECT. All model training were completed in a Python environment. CosinorPy was used to fit proteins to cosine curves and provide their geometrical information, including amplitude, mesor, and acrophase. For downstream analysis, we used compareRhythms and circaCompare packages to analyze disparities between AD and control subjects. WGCNA was used to construct co-expression networks, and hub proteins were identified with cytoHubba in Cytoscape. Gene ontology enrichment analysis was conducted using GSEApy.

## Supplemental information

Document S1. Figures S1–S15 and Table S1–S4

Data S1. Spreadsheet of rhythmic proteins identified in individual datasets and shared across tissues, regions and species
