# Supplementary material for: PROTECT: Protein circadian time prediction using unsupervised learning
Source: iScience. 2025 Sep 22;28(10):113605. doi: 10.1016/j.isci.2025.113605 (PMC12539262; doi:10.1016/j.isci.2025.113605)
Supplement: Document S1. Figures S1–S15 and Table S1–S4 [file mmc1.pdf]

iScience, Volume *28*

Supplemental information

PROTECT: Protein circadian time prediction using unsupervised learning

Aram Ansary Ogholbake and Qiang Cheng

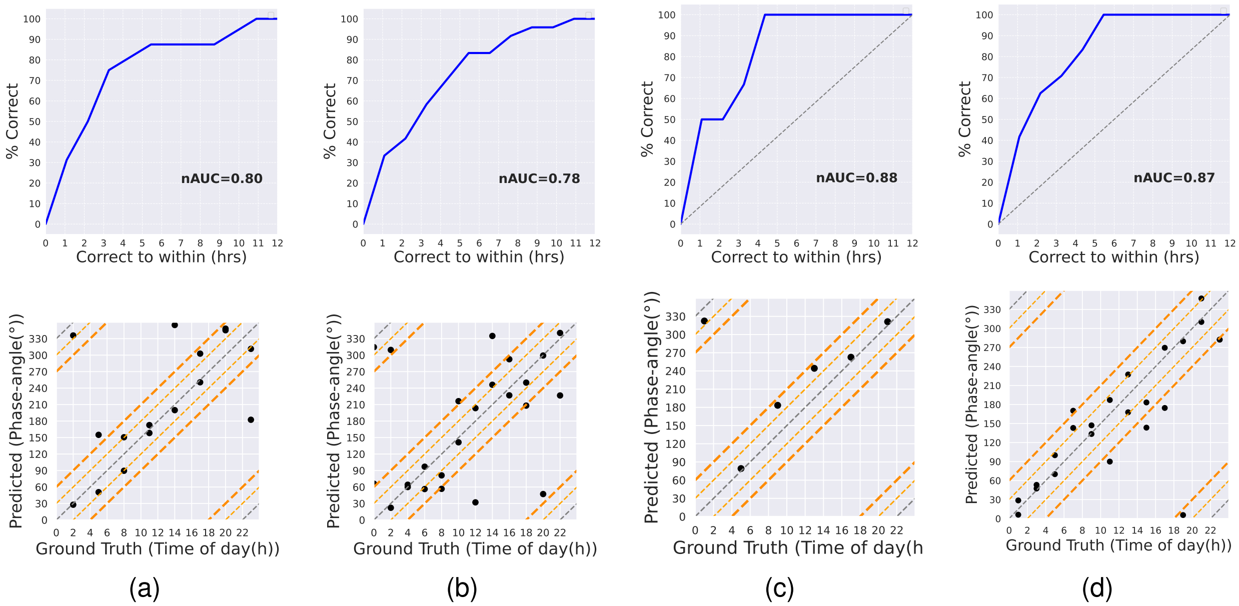

Figure S1: Accuracy of PROTECT on (a) mouse liver and (b) mouse BAT, (c) Arabidopsis thaliana plants and (d) Neurospora crassa. The top row shows ROC curves where the y-axis shows the fraction of correctly predicted samples, and the x-axis shows the size of errors. The bottom row shows the scatter plots of predictions vs ground truth.

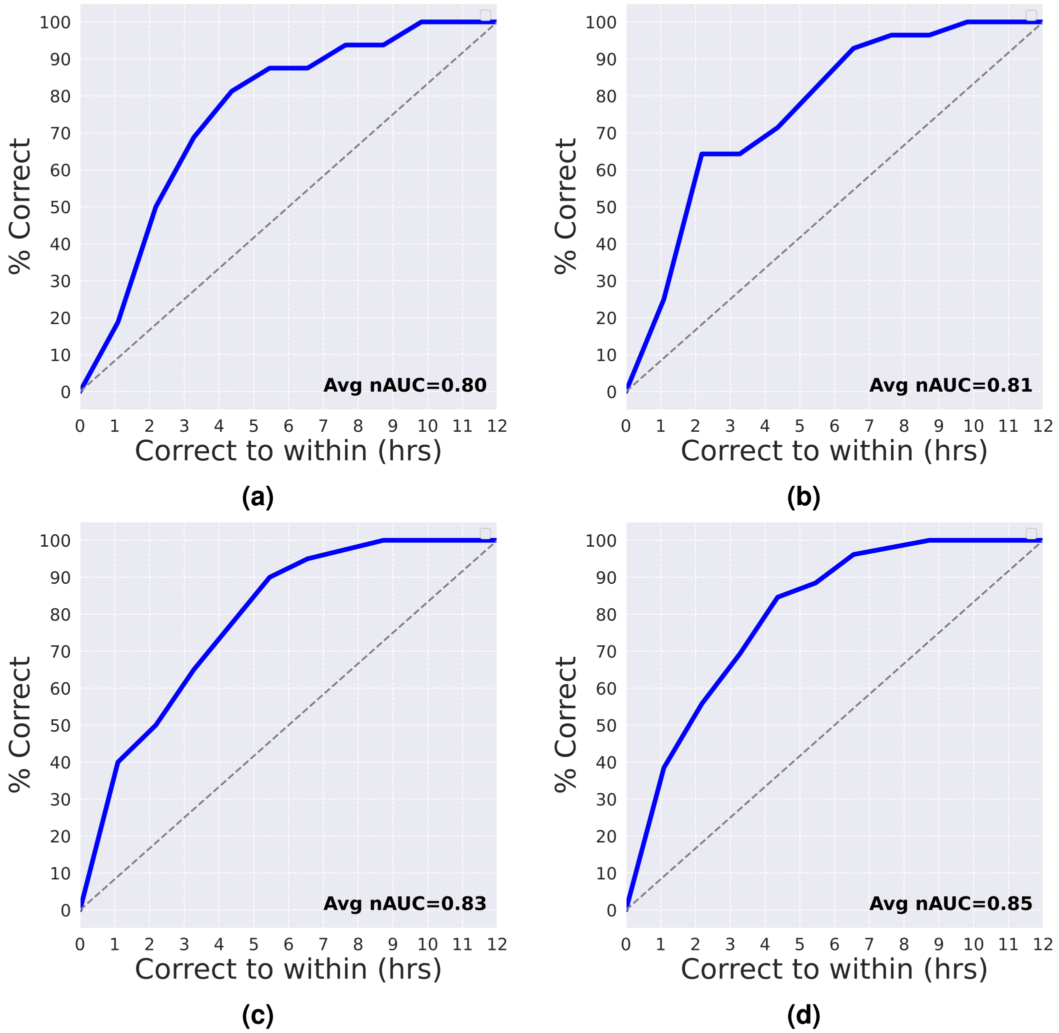

Figure S2: Results on mouse liver dataset of Wang et al. [29] using less number of samples: (a) using 4 samples, (b) using 7 samples, (c) using 10 samples, and (d) using 13 samples.

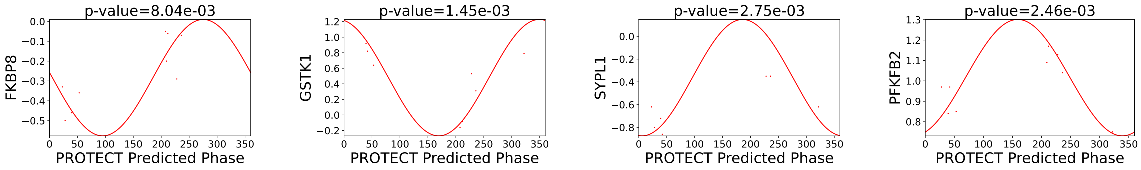

Figure S3: Plots of four randomly chosen proteins known to be rhythmic in thyroid tissue using predicted phases by PROTECT. The y-axis represents protein expression levels, and the x-axis represents the predicted phases (in degrees) as determined by PROTECT.

**Dataset**	**PROTECT nAUC**	**CYCLOPS nAUC**	**Relative Improvement**
O. tauri	0.94	0.76	0.24
Cartilage	0.83	0.65	0.28
Mouse Liver 1	0.84	0.59	0.42
Mouse Liver 2	0.90	0.64	0.41
Mouse Liver 3	0.80	0.53	0.51
Mouse BAT	0.78	0.66	0.18
Human Plasma	0.82	0.73	0.12
Arabidopsis thaliana	0.88	0.73	0.21
Mouse Liver (transcriptomic) [30]	0.95	0.85	0.12

Table S1: nAUC comparison of PROTECT and CYCLOPS on time-labeled proteomic datasets and a mouse liver transcriptomic dataset. PROTECT consistently outperformed CYCLOPS across diverse tissues and species, including cell, mouse, human, and plant datasets. The relative improvement is defined as $\frac{\text{PROTECT nAUC} - \text{CYCLOPS nAUC}}{\text{CYCLOPS nAUC}}$, and is also reported. Notably, PROTECT does not rely on prior knowledge, in contrast to CYCLOPS, which uses proteins corresponding to seed rhythmic genes when available.

(a) Summary of Rhythmicity Classification (compareRhythms)

**Category**	**Number of Proteins**
Lost rhythmicity in AD	316
Gained rhythmicity in AD	272
Rhythmic in both (no change)	43
Rhythmic in both (phase/amplitude changed)	407

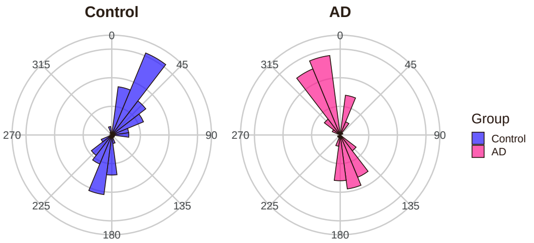

(b) Peak time distribution

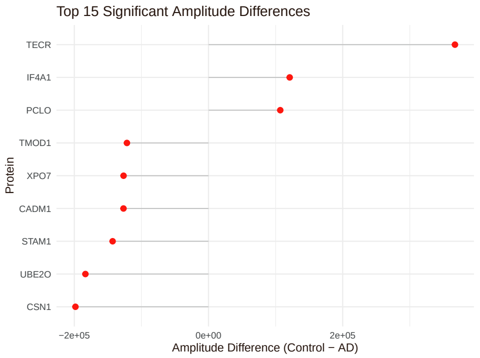

(c) Top 15 amplitude differences

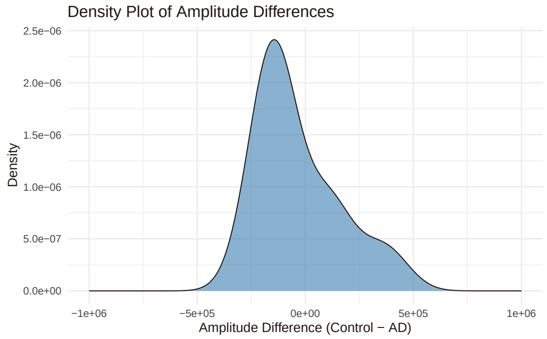

(d) Density of amplitude differences

Figure S4: Disparities between control and AD subjects in the parietal association cortex. (a) Summary of rhythmicity classification from compareRhythms. (b–d) Visualization of peak time distribution, top proteins with high and significant amplitude changes, and overall amplitude difference density using PROTECT predicted phases.

(a) Summary of Rhythmicity Classification (compareRhythms)

Category	Number of Proteins
Lost rhythmicity in AD	81
Gained rhythmicity in AD	80
Rhythmic in both (no change)	5
Rhythmic in both (phase/amplitude changed)	1404

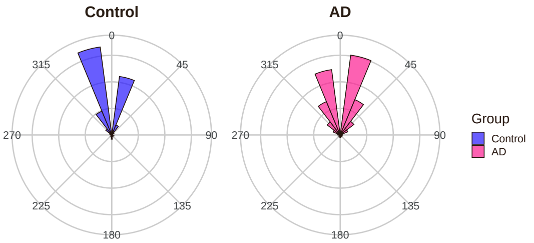

(b) Peak time distribution

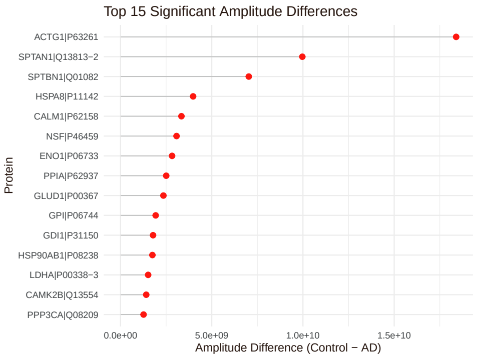

(c) Top 15 amplitude differences

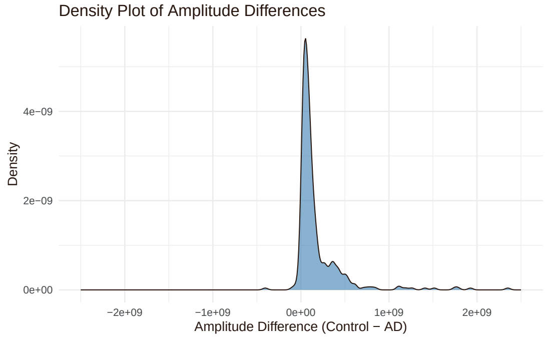

(d) Density of amplitude differences

Figure S5: Disparities between control and AD subjects in DLPFC. (a) Summary of rhythmicity classification from compareRhythms. (b–d) Visualization of peak time distribution, top proteins with high and significant amplitude changes, and overall amplitude difference density using PROTECT predicted phases.

Dataset	PROTECT nAUC (w/o time labels)	TimeSignature nAUC (with time labels)	ZeitZeiger nAUC (with time labels)
Mouse liver Proteomic	0.94	0.95	0.92
Mouse liver Transcriptomic	0.97	0.98	0.96

Table S2: Comparison of averaged nAUCs between PROTECT and supervised methods (TimeSignature and ZeitZeiger) on proteomic and transcriptomic datasets.

Category	CT vs AD	CT vs MCI	AD vs MCI
Lost rhythmicity (in the second group)	14	18	18
Gained rhythmicity (in the second group)	4	4	19
Rhythmic in both (no change)	273	88	1
Rhythmic in both (phase/amplitude changed)	163	339	411
**Shared rhythmic proteins across all groups**	**349**		

Table S3: Summary of rhythmicity classification in urine proteome across three comparisons using compareRhythms. The final row indicates the number of rhythmic proteins shared across all comparison groups.

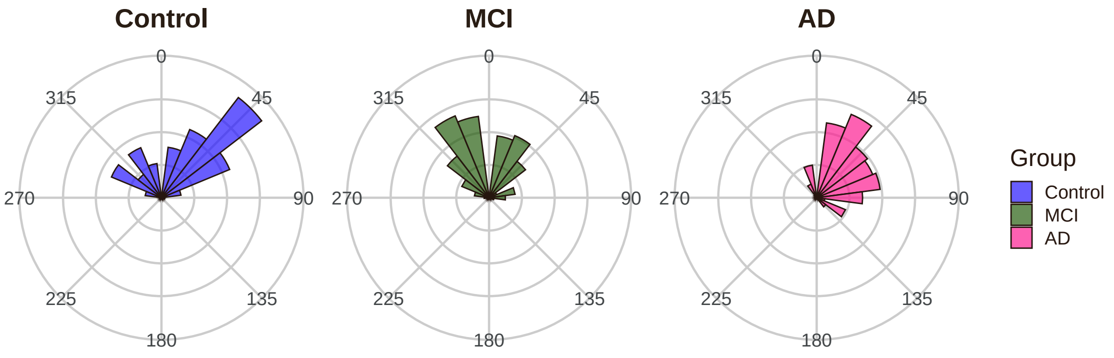

Figure S6: Peak time distribution across Control, MCI, and AD groups in urine data.

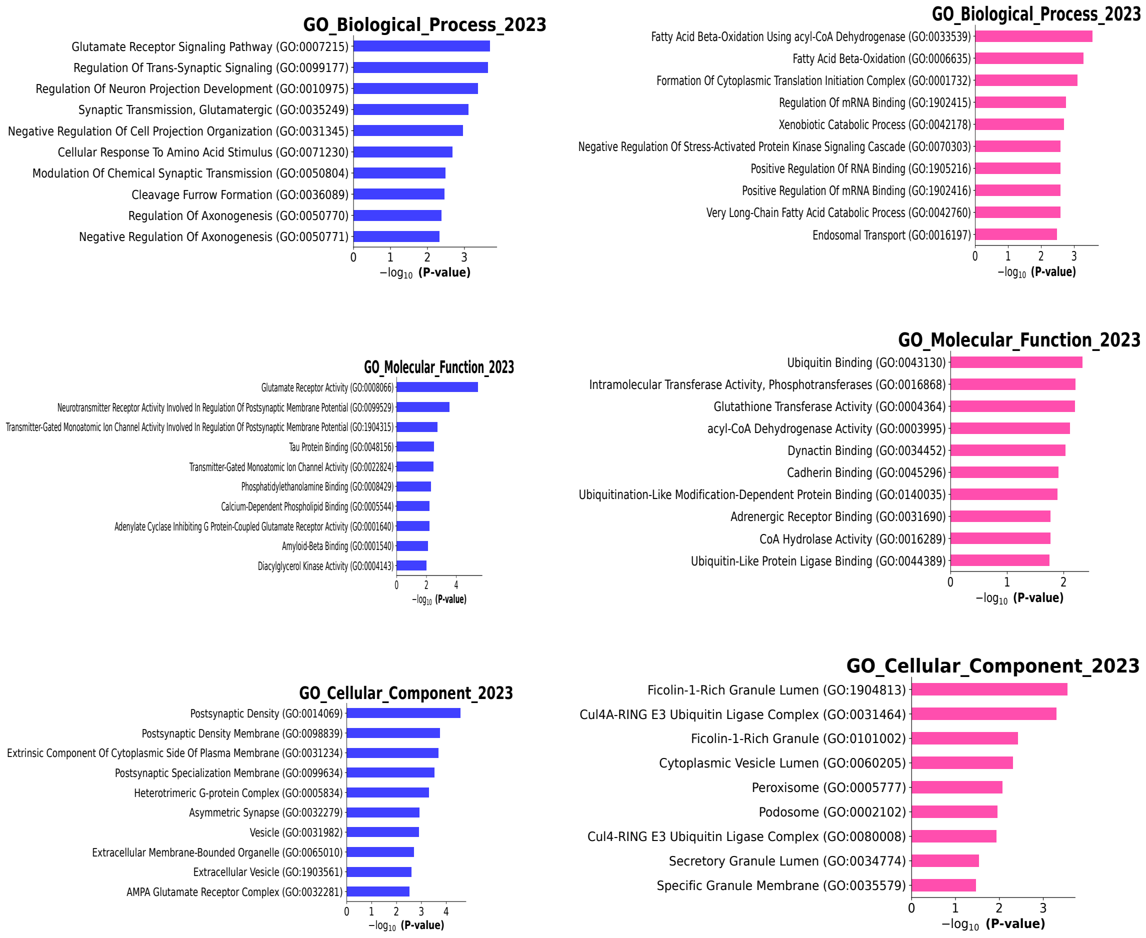

Figure S7: Gene Ontology enrichment analysis of proteins that lose rhythmicity in AD (blue), and in proteins that gain rhythmicity in AD subjects (pink) on parietal association cortex.

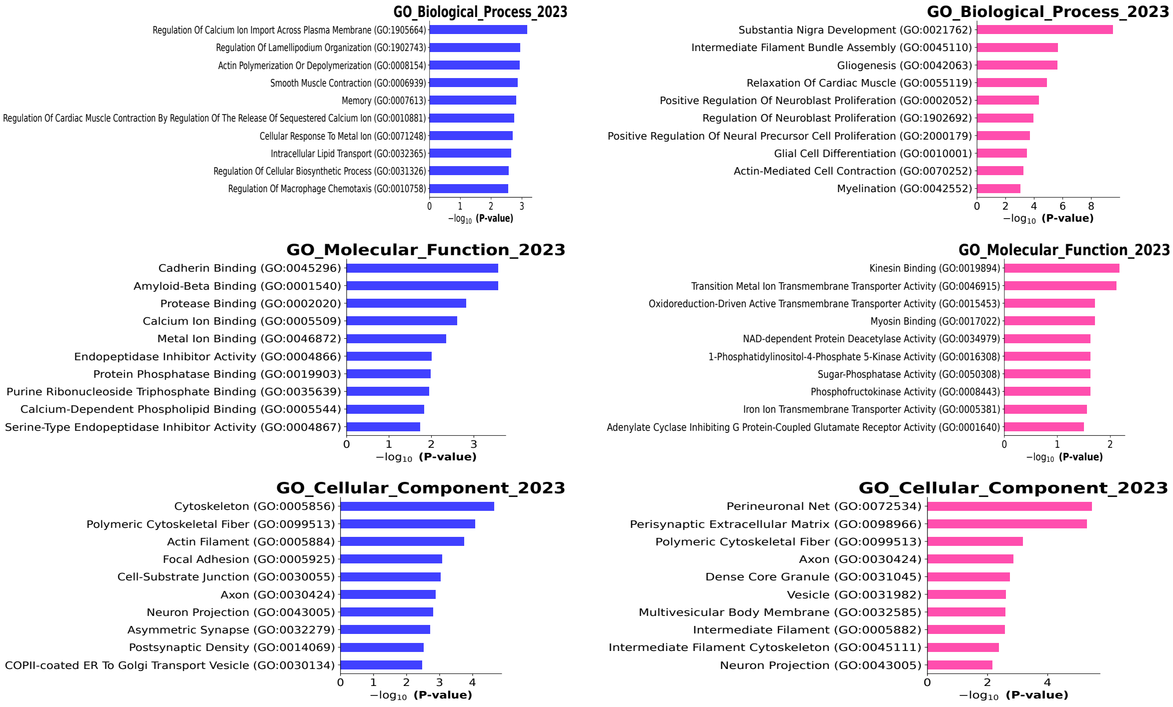

Figure S8: Gene Ontology enrichment analysis of proteins that lose rhythmicity in AD (blue), and in proteins that gain rhythmicity in AD subjects (pink) in DLPFC brain region.

DLPFC Region	Parietal Association Cortex Region
CNBP	CA198
COPS8	LIMC1
PRNP	BCAS1
VBP1	ERMIN
SEC13	TENA
TMOD1	NINJ2
COQ5	BCAS1.1
CPT2	GAPR1
GFAP	G3BP2
PPP3CB	GCP3
TIPRL	RHOG
EIF3E	CRYAB
ADRM1	LYPA1
C19orf52	PLCL2
SF1	EMC3
F13A1	VP9D1
GATM	ENPP2
MAPK3	TBB4B
SLC8A1	LPPRC
CDIPT	F8W059

Table S4: First 20 Hub Proteins in DLPFC and parietal association cortex.

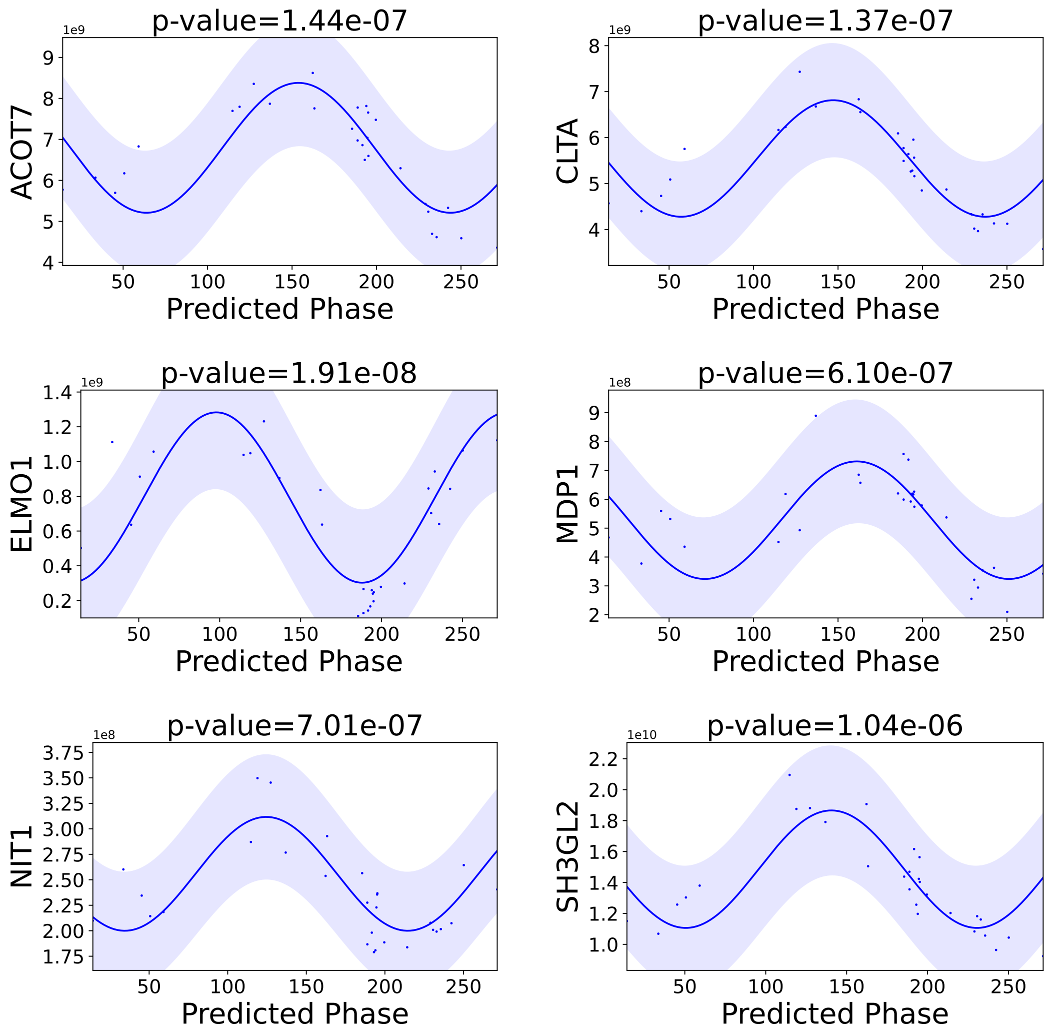

Figure S9: Ultradian proteins found in temporal cortex with period of 12 hours.

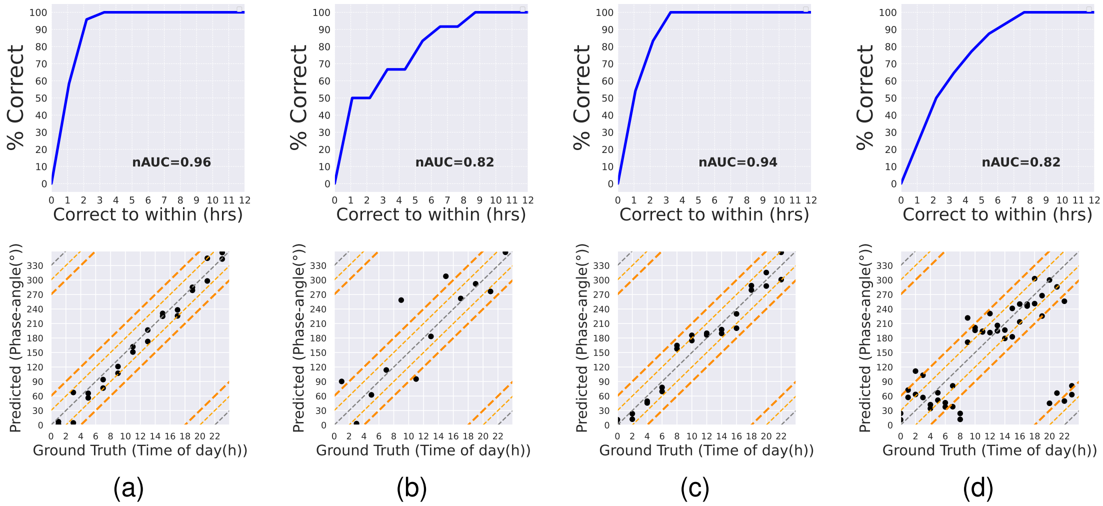

Figure S10: Accuracy of PROTECT on transcriptomic and metabolomic data. (a) mouse liver transcriptomic, (b) baboon amygdala transcriptomic, (c) mouse kidney transcriptomic, and (d) mouse liver metabolomic . The top row shows ROC curves where the y-axis shows the fraction of correctly predicted samples, and the x-axis shows the size of errors. The bottom row shows the scatter plots of predictions vs ground truth.

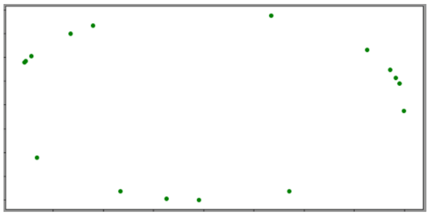

Figure S11: Mouse liver encoded data in pre-training stage.

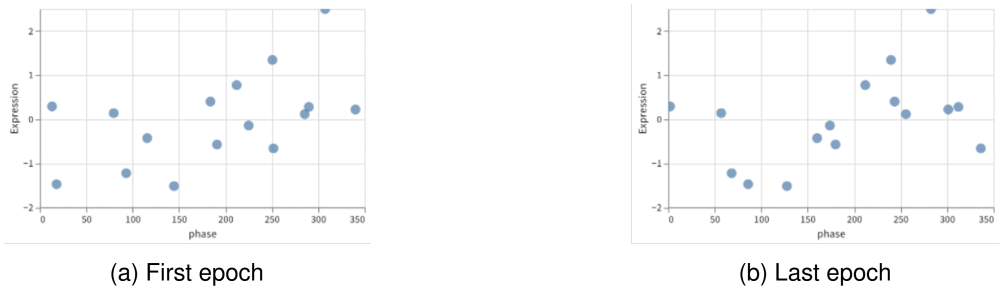

Figure S12: Training progress on a random protein in mouse liver data during fine-tuning stage.

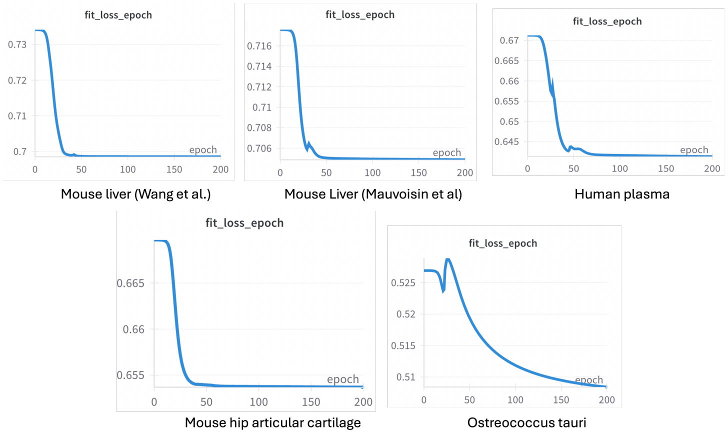

Figure S13: Convergence results on time labeled datasets.

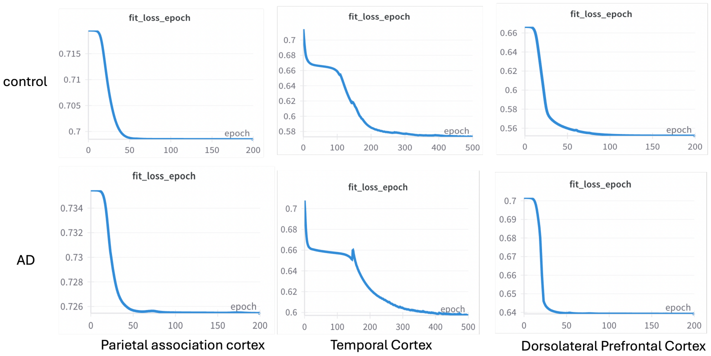

Figure S14: Convergence results on brain datasets.

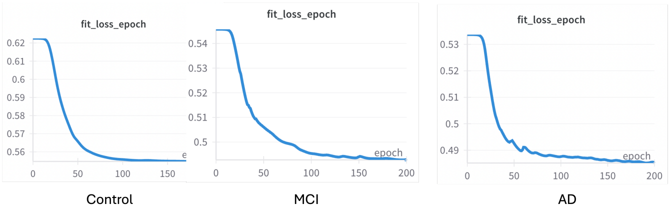

Figure S15: Convergence results on urine dataset.
